# Supplementary material for: Live tumor imaging shows macrophage induction and TMEM-mediated enrichment of cancer stem cells during metastatic dissemination
Source: Nat Commun. 2021 Dec 15;12:7300. doi: 10.1038/s41467-021-27308-2 (PMC8674234; doi:10.1038/s41467-021-27308-2)
Supplement: Supplementary file 1 — Supplementary Information [file 41467_2021_27308_MOESM1_ESM.pdf]

Supplementary Information

Live tumor imaging shows macrophage-induction and TMEM-mediated enrichment of cancer stem cells during metastatic dissemination

Ved P Sharma[1,2], Binwu Tang[4], Yarong Wang[1,2,3], Camille L Duran[1], George S Karagiannis[1,3], Emily A Xue[1], David Entenberg[1,2,3], Lucia Borriello[1], Anouchka Coste[1,7], Robert J Eddy[1], Gina Kim[1], Xianjun Ye[1], Joan G Jones[1,3,6,8], Eli Grunblatt[9], Nathan Agi[9], Sweta Roy[9], Gargi Bandyopadhyaya[9], Esther Adler[10], Chinmay R Surve[1,3], Dominic Esposito[5], Sumanta Goswami[1,9], Jeffrey E Segall[1], Wenjun Guo[11,12], John S Condeelis[1,2,3,7,*], Lalage M. Wakefield[4,*], Maja H Oktay[1,2,3,6,*]

[1] Department of Anatomy and Structural Biology, Albert Einstein College of Medicine, Bronx, NY, USA
[2] Gruss-Lipper Biophotonics Center, Albert Einstein College of Medicine, Bronx, NY, USA
[3] Integrated Imaging Program, Albert Einstein College of Medicine, Bronx, NY, USA
[4] Laboratory of Cancer Biology and Genetics, National Cancer Institute, Bethesda, MD USA
[5] Protein Expression Laboratory, Frederick National Laboratory for Cancer Research, Frederick MD, USA
[6] Department of Pathology, Albert Einstein College of Medicine, Bronx, NY, USA
[7] Department of Surgery, Albert Einstein College of Medicine, Bronx, NY, USA
[8] Department of Epidemiology and Population Health, Albert Einstein College of Medicine, Bronx, NY, USA
[9] Department of Biology, Yeshiva University, New York, NY, USA
[10] Department of Pathology, NYU Langone Medical Center, NY, USA
[11] Department of Cell Biology, Albert Einstein College of Medicine, Bronx, NY, USA
[12] Ruth L. and David S. Gottesman Institute for Stem Cell and Regenerative Medicine Research, Albert Einstein College of Medicine, Bronx, NY, USA

* Co-senior and corresponding authors:
john.condeelis@einsteinmed.org, wakefiel@dce41.nci.nih.gov, moktay@montefiore.org

Table of Contents

Figure S1: Characterization of SORE6 stemness reporter. 3

Figure S2: Characterization of FLAG antibody for identifying SORE6+ stem cells. 5

Figure S3: Optimization of imaging conditions for identifying SORE6+ cancer stem cells during intravital imaging in living mouse. 6

Figure S4: SORE6+ CSCs exhibit the slow migratory, invadopodium rich phenotype both in vivo and in vitro. 9

Figure S5: SORE6 reporter marks a minority cell population that is enriched in Sox9 stemness transcription factor in PyMT-derived Met-1 cells. 10

Figure S6: Macrophage contact induces stemness (SORE6 positivity) in multiple breast cancer cell lines 13

Figure S7: DAPT inhibits Notch signaling in vivo. 14

Figure S8: Additional data for Figures 6 and 7 16

Supplementary Table 1: Primer pair sequences for qRT-PCR of stem cell transcription factors 17

a

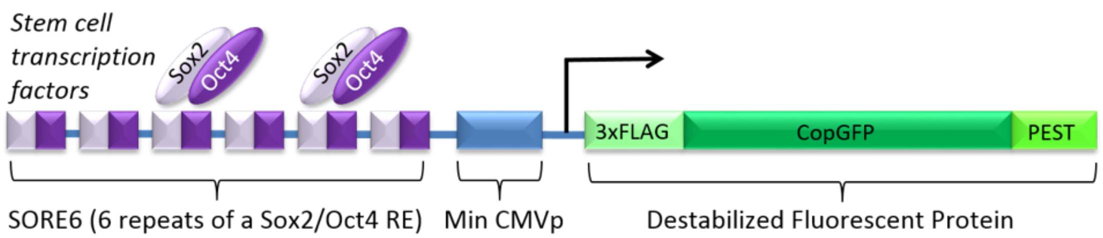

b

		Tumor incidence at day 98 at different starting cell inocula (# tumors/#sites)				Cancer stem cell frequency	Fold enrichment of CSCs in SORE6+ population	Chisq p-value for difference in frequency
Initial cell inoculum (# cells/site)		7000	5000	1000	250			
Cells implanted	SORE6+	5/6	5/6	2/6	1/6	**1 in 2974**	**7.8**	**0.00033**
	SORE6-	2/6	1/6	0/6	0/6	**1 in 23186**		

c

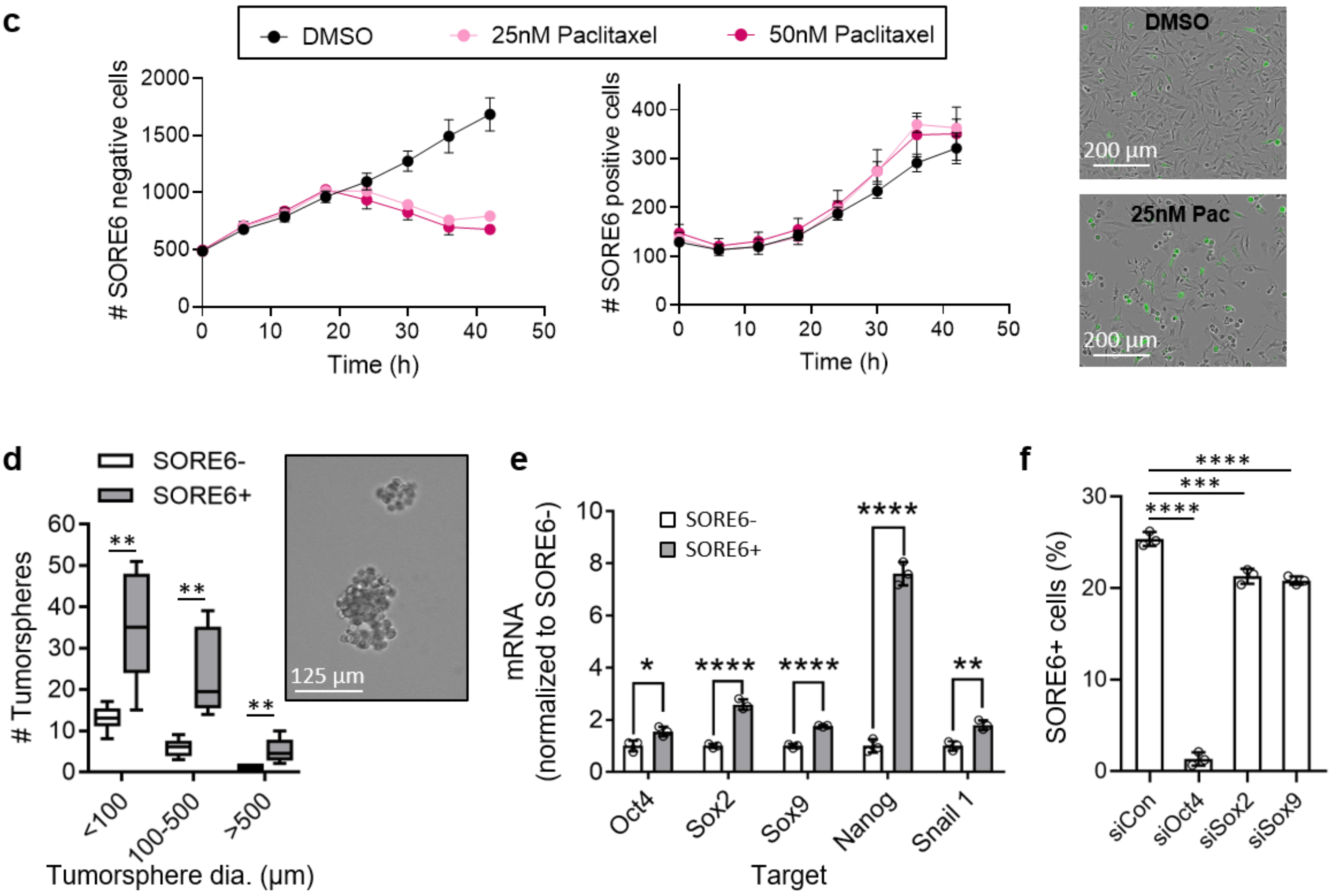

g

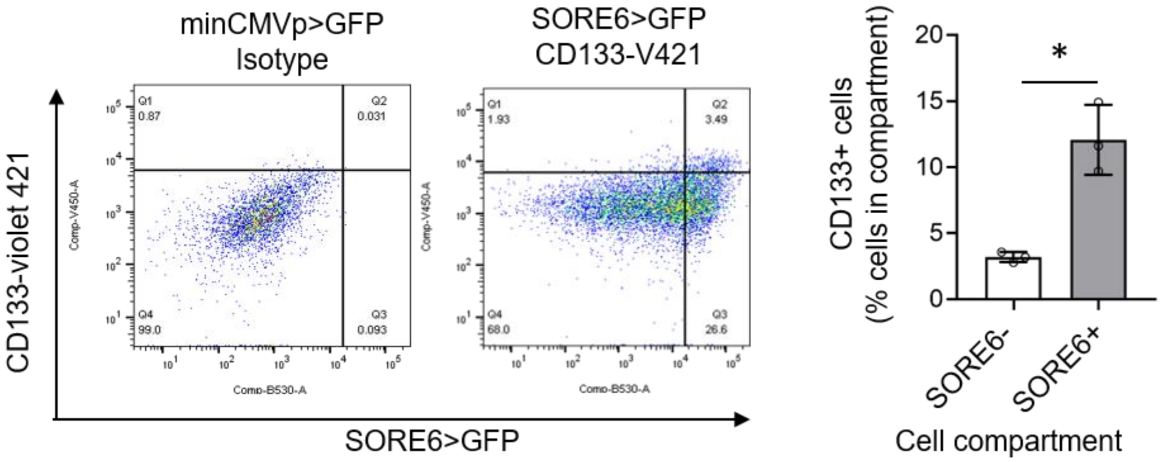

h

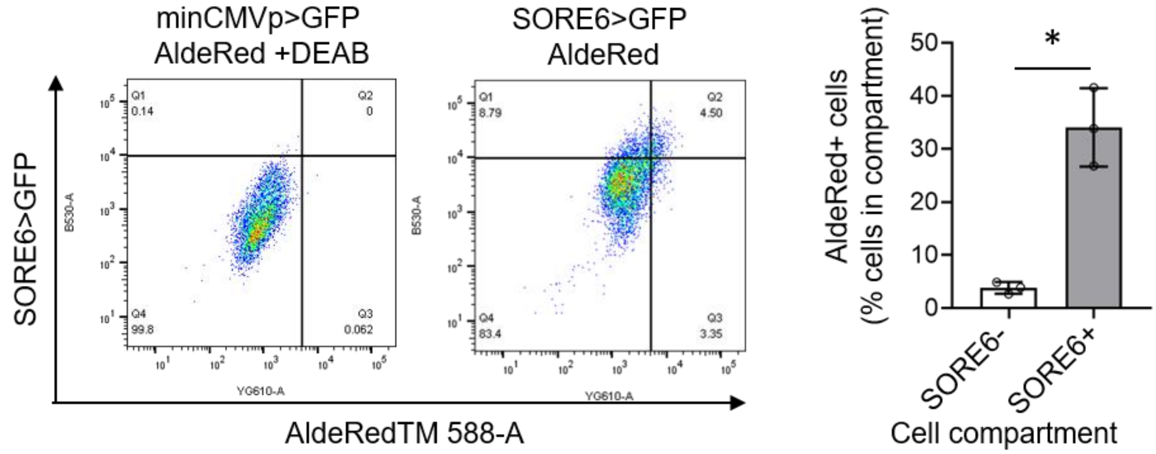

i

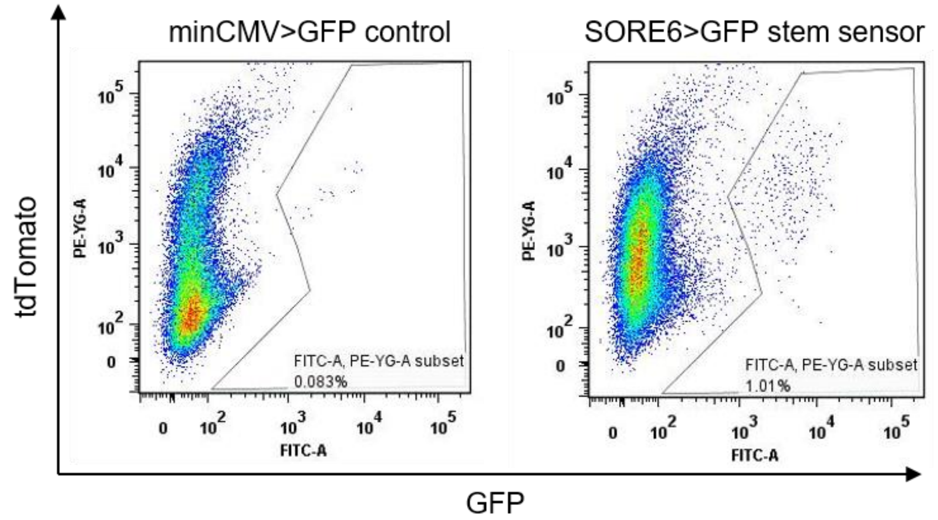

Figure S1: Characterization of SORE6 stemness reporter.

- **a.** SORE6 stem cell reporter schematic. Three tandem FLAG epitopes were inserted before the dsCopGFP reporter for detection of SORE6+ cells in fixed tissue using FLAG antibody. This stem cell reporter construct is referred to as “SORE6>GFP” throughout.

b. In vivo limiting dilution assay showing nearly 8-fold enrichment for tumor-initiating ability of SORE6+ MDA-MB-231 cells compared to SORE6- MDA-MB-231 cells in SCID mice. 95% confidence intervals for the cancer stem cell frequency are 1 in 5608 to 1 in 1577 for SORE6+ cells, and 1 in 71505 to 1 in 7518 for SORE6- cells.

c. SORE6-positive cells are resistant to chemotherapeutics. Line plots are shown with mean ± SD (n=3). Images on the right show SORE6- (no green color) and SORE6+ (with green color) under DMSO and Paclitaxel (Pac) conditions at t=42h.

d. SORE6+ MDA-MB-231 cells are enriched for tumorsphere-forming ability, with representative tumorsphere image. The boxes indicate 25th-75th percentile interquartile range (IQR) and central line indicates the median. Top and bottom whiskers were plotted using Tukey method and extend to 75th percentile+1.5*IQR and 25th percentile-1.5*IQR, respectively. Multiple unpaired two-sided t-tests, n=6, p-values: **(<100 µm) =0.00272, **(100-500 µm) =0.00213 and **(>100 µm) =0.00756.

e. qRT-PCR of stem cell and EMT master transcription factor transcripts in MDA-MB-231 cells after FACS sorting into SORE6+ and SORE6- populations. Data are mean ± SD (n=3); unpaired two-sided t-tests; p-values: *(Oct4) =0.0256, ****(Sox2) =0.0002, ****(Sox9) =0.0001, ****(Nanog) =0.00002, **(Snail1) =0.0052.

f. Knockdown of master stem cell transcription factors reduces or eliminates SORE6+ cells as assessed by flow cytometry. Data are mean ± SD (n=3). ****p =0.0001, ***p =0.0002, Two-sided Dunnet’s multiple comparisons test vs siCon.

g. Enrichment of SORE6+ cells in CD133+ vs CD133- populations of MDA-MB-231 cells assessed by flow cytometry. Results are mean ± SD (n=3), paired two-tailed Student’s t-test, *p =0.024.

h. Enrichment of SORE6+ cells in AldeRed+ (ALDH active) vs AldeRed- cell populations. Results are mean ± SD (n=3), paired two-tailed Student’s t-test, *p =0.014.

i. FACS analysis showing that the SORE6 reporter identifies a minority population in MDA-MB-231 tumors in SCID mice of ~ 1%.

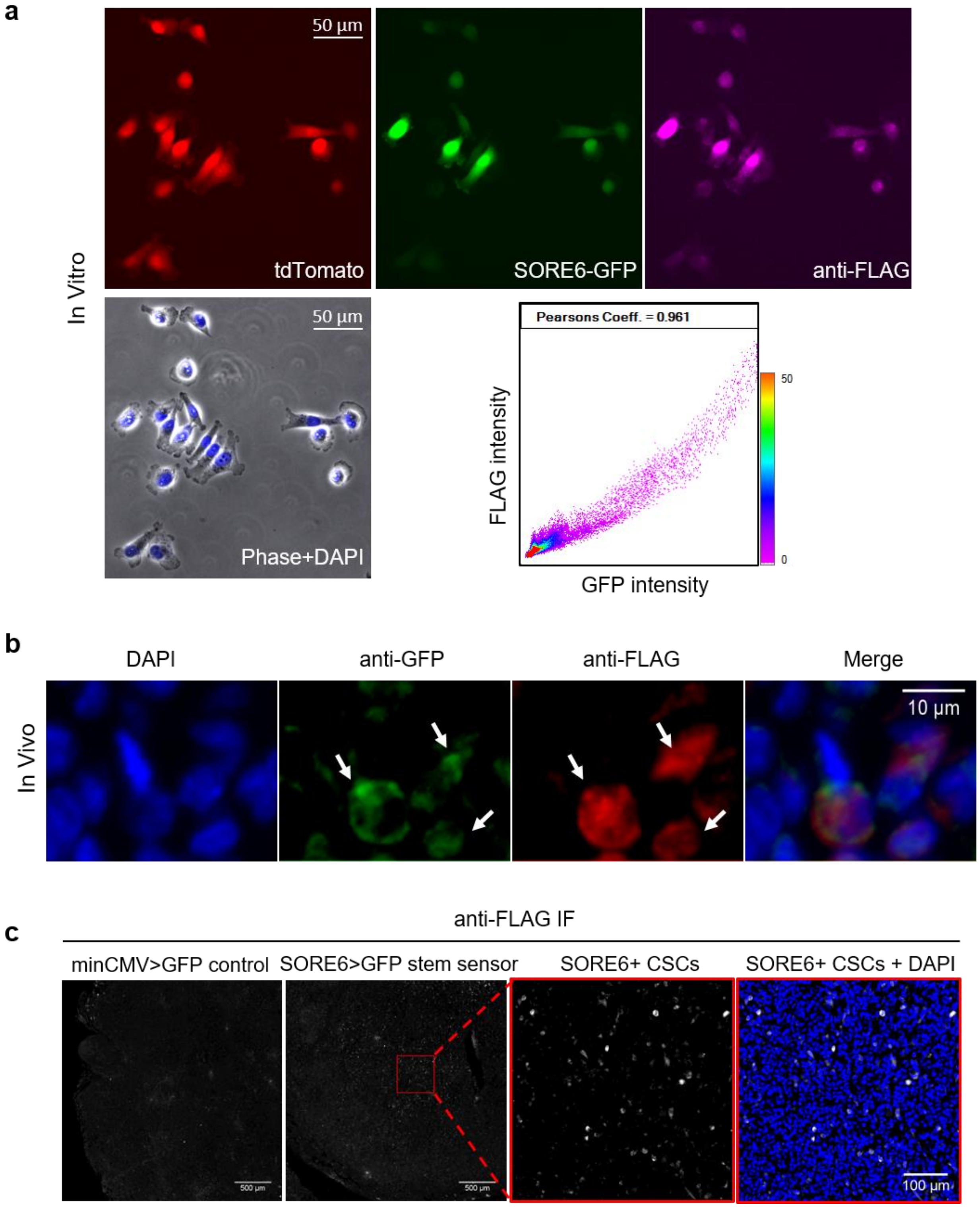

Figure S2: Characterization of FLAG antibody for identifying SORE6+ stem cells.

a. MDA-MB-231 cells expressing tdTomato volume marker and containing the SORE6>GFP reporter construct were plated on glass-bottom dishes, fixed and stained with FLAG antibody to detect the FLAG tag on the dsCopGFP. Antibody staining shows the presence of FLAG in

SORE6-GFP positive cells. Scatter plot shows a strong positive correlation between GFP and FLAG signals to identify the SORE6+ tumor cells (Pearson's coefficient = 0.96).

b. SORE6>GFP sensor expressing MDA-MB-231 primary tumor FFPE sections were stained with anti-GFP and anti-FLAG antibodies and show the presence of FLAG in SORE6-GFP positive cells in vivo (white arrows). Similar results were obtained in n=2 mice independently.

c. *In situ* immunofluorescence anti-FLAG staining identifies minority population of SORE6+ cancer stem cells in fixed tissues in nude mouse host. Left panel: tdTomato-MDA-MB-231 – minCMV>GFP vector control tumor tissue, right three panels: tdTomato-MDA-MB-231-SORE6>GFP tumor tissue. SORE6 expressing CSCs stained with anti-FLAG are shown in white. Similar results were obtained in n=3 mice independently.

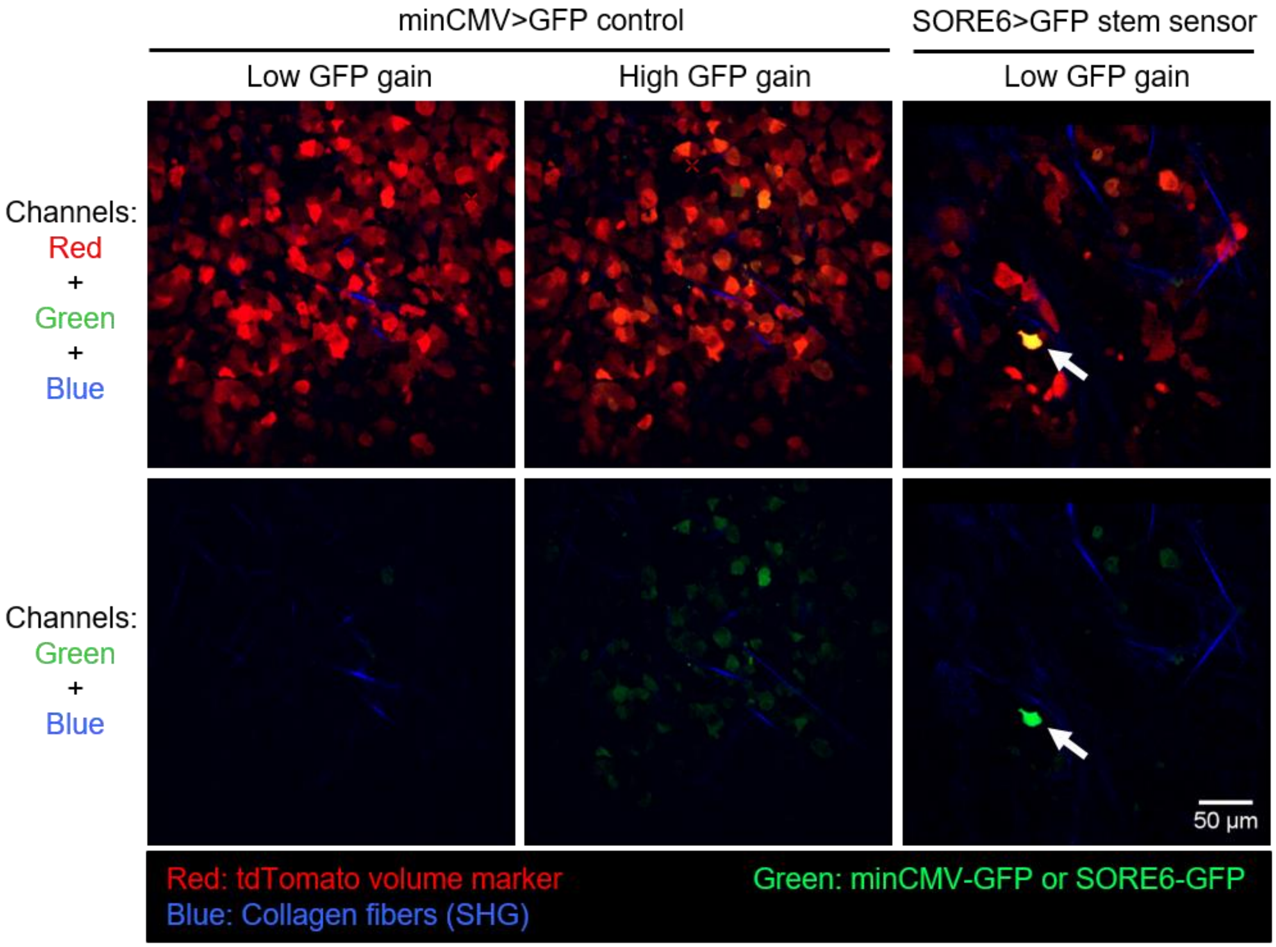

Figure S3: Optimization of imaging conditions for identifying SORE6+ cancer stem cells during intravital imaging in living mouse.

Mice with tumor cells expressing minCMV>GFP control plasmid were imaged at low GFP gain settings (left panels) to set the GFP background threshold. High GFP gain image of the same field for minCMV>GFP (middle panels) clearly show low background GFP levels in tumor cells. All intravital imaging in mice was performed at low GFP gain setting and shows minor population of cancer stem cells (right panels, white arrow) in tumors expressing SORE6 reporter. Similar results were obtained in n=10 mice independently.

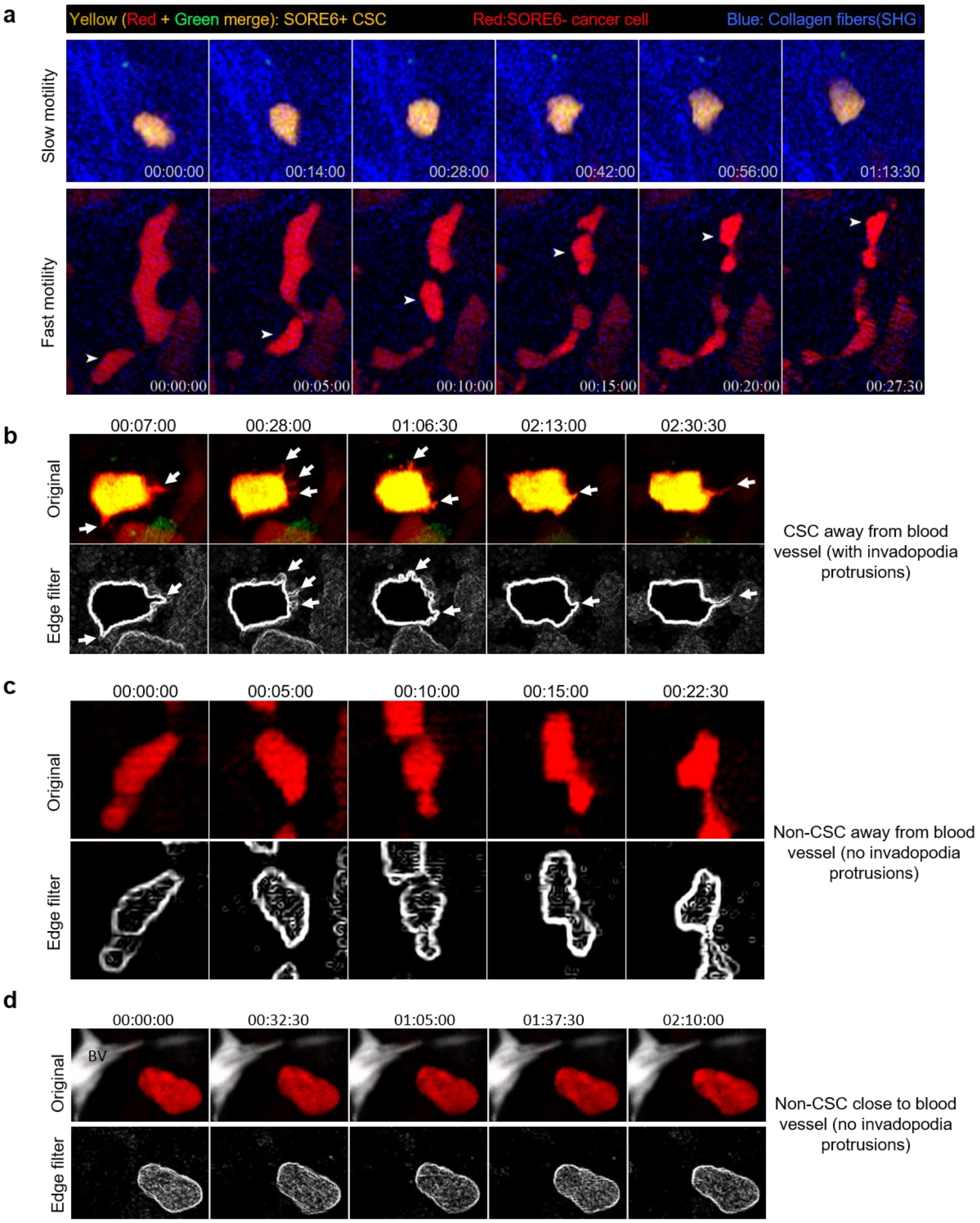
a
Yellow (Red + Green merge): SORE6+ CSC
Red:SORE6- cancer cell
Blue: Collagen fibers(SHG)
Slow motility
00:00:00
00:14:00
00:28:00
00:42:00
00:56:00
01:13:30
Fast motility
00:00:00
00:05:00
00:10:00
00:15:00
00:20:00
00:27:30
b
00:07:00
00:28:00
01:06:30
02:13:00
02:30:30
Original
Edge filter
CSC away from blood vessel (with invadopodia protrusions)
c
00:00:00
00:05:00
00:10:00
00:15:00
00:22:30
Original
Edge filter
Non-CSC away from blood vessel (no invadopodia protrusions)
d
00:00:00
00:32:30
01:05:00
01:37:30
02:10:00
BV
Original
Edge filter
Non-CSC close to blood vessel (no invadopodia protrusions)

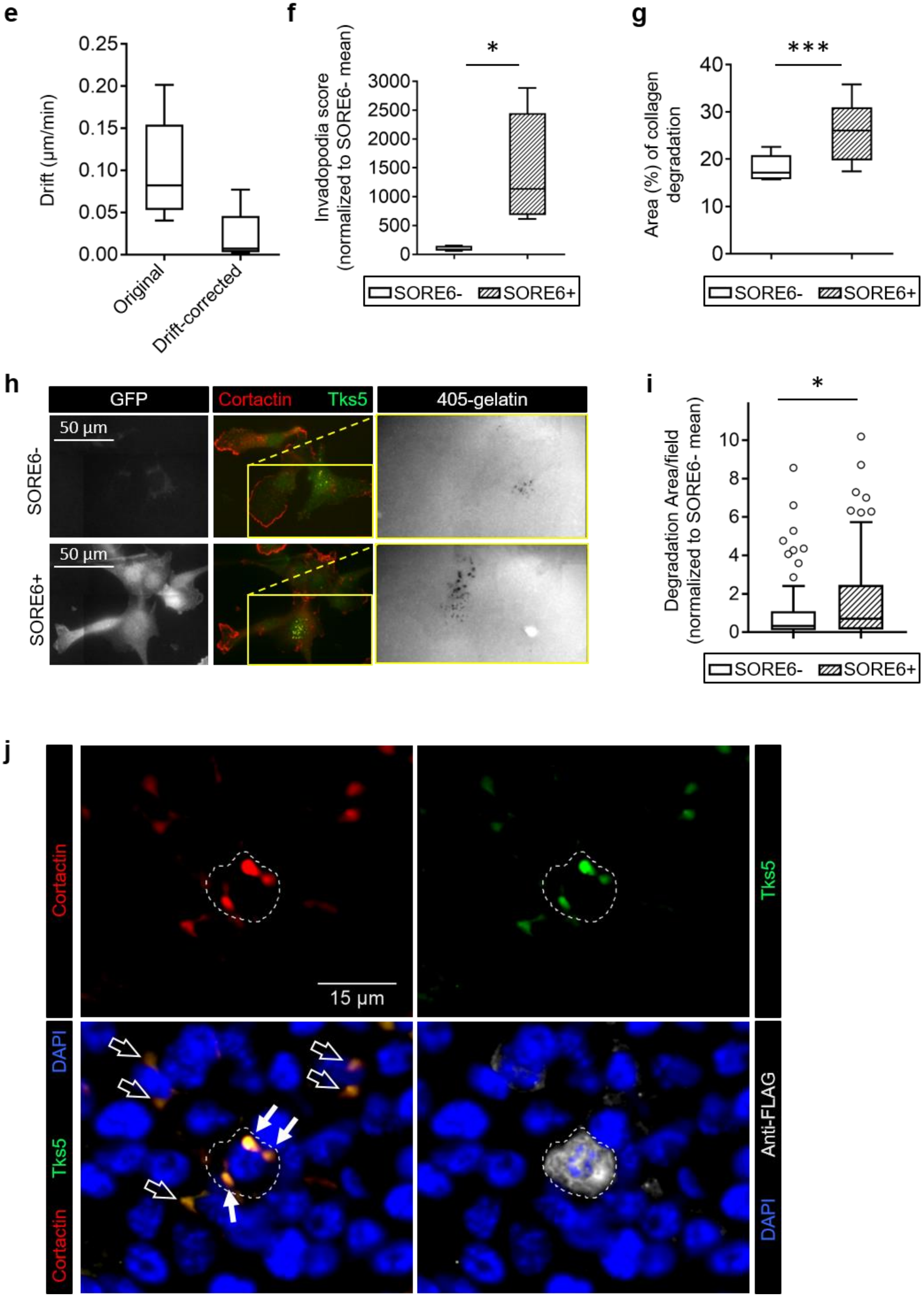
e
Drift (µm/min)
0.25
0.20
0.15
0.10
0.05
0.00
Original
Drift-corrected
f
Invadopodia score (normalized to SORE6- mean)
*
3000
2500
2000
1500
1000
500
0
SORE6-
SORE6+
g
Area (%) of collagen degradation
***
40
30
20
10
0
SORE6-
SORE6+
h
GFP
Cortactin Tks5
405-gelatin
50 µm
SORE6-
50 µm
SORE6+
i
Degradation Area/field (normalized to SORE6- mean)
*
10
8
6
4
2
0
SORE6-
SORE6+
j
Cortactin
Tks5
15 µm
DAPI
Tks5
Cortactin
Anti-FLAG
DAPI

Figure S4: SORE6+ CSCs exhibit the slow migratory, invadopodium rich phenotype both in vivo and in vitro.

- **a.** Panels from Movies 1 and 2 depicting slow and fast motility phenotypes of CSC and non-CSC (related to Fig 2a).
- **b.** Panels from Movie 6 showing a SORE6+ CSC, which is away from the blood vessel, displaying dynamic invadopodial protrusions (white arrows) coming out in all directions. Bottom panels show the dynamic invadopodial protrusions (white arrows) more clearly after applying the edge filter.
- **c.** Panels from Movie 7, for SORE6- fast migrating cells show amoeboid movement without spike shaped invasive protrusions. Bottom panels show images with the edge filter.
- **d.** Panels from Movie 8, for SORE6- cell close to blood vessel (BV: white) show amoeboid movement without spike shaped invasive protrusions. Bottom panels show images with the edge filter.
- **e.** Quantification of XY drift in time-lapse intravital movies of live breast tumor tissue before and after correcting for the drift using HyperStackReg plugin (Movie 9, see Methods). n=5 fields of 340x340 μm^2 from 3 mice.
- **f.** Invadopodia score quantification in non-CSCs and SORE6+ CSCs using a manual scoring method. n=5 fields at 40X magnification from 4 tumors; paired two-tailed Student's t-test, *p =0.032.
- **g.** Quantification of collagen degradation area in non-CSC and CSC areas using a manual scoring method. n=6 fields at 40X magnification from 4 SORE6 tumors; paired two-tailed Student's t-test, ***p =0.0095.
- **h.** Representative images showing in vitro invadopodia-associated degradation in sorted SORE6- and SORE6+ cells. Yellow rectangles in middle panels indicate the zoomed area shown for the 405-gelatin images. Black areas in the 405-gelatin channel depict degraded matrix.
- **i.** Quantification of matrix degradation by SORE6- and SORE6+ cells. n=85 fields of 110x110 μm^2 at 60X magnification for SORE6- and n=61 fields of 110x110 μm^2 at 60X magnification for SORE6+ samples; unpaired two-tailed Student's t-test, *p =0.0187.
- **j.** A zoomed-out view of the image in Fig. 2h. Solid and empty arrows mark Cortactin and Tks5 positive invadopodia in CSC (FLAG-positive cell) and non-CSCs (other FLAG-negative cells), respectively. Similar results were obtained in n=4 mice independently.

e, f, g, i The boxes indicate 25th-75th percentile interquartile range (IQR) and central line indicates the median. Top and bottom whiskers were plotted using Tukey method and extend to 75th percentile+1.5*IQR and 25th percentile-1.5*IQR, respectively. Points below and above the whiskers are drawn as individual dots.

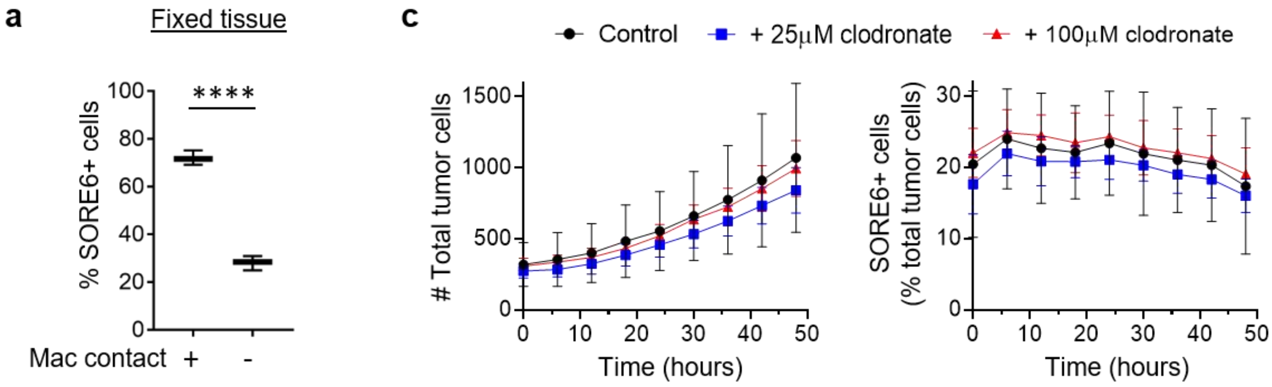

b

Number of SORE6+ cells

Control				Clodronate			
Field #	Day 0	Day 1	Day 2	Field #	Day 0	Day 1	Day 2
1	21	13	15	1	12	6	4
2	18	18	17	2	7	5	3
3	16	12	13	3	11	6	2
4	14	13	13	4	11	6	1
5	14	15	14	5	8	8	6
6	14	13	11	6	35	15	10
7	24	31	26	7	21	16	11

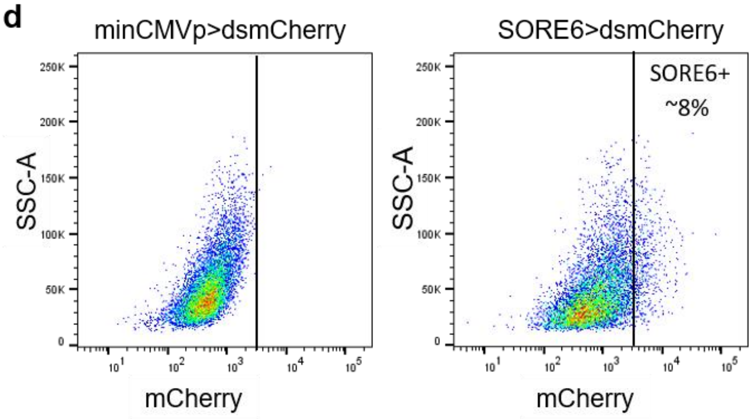

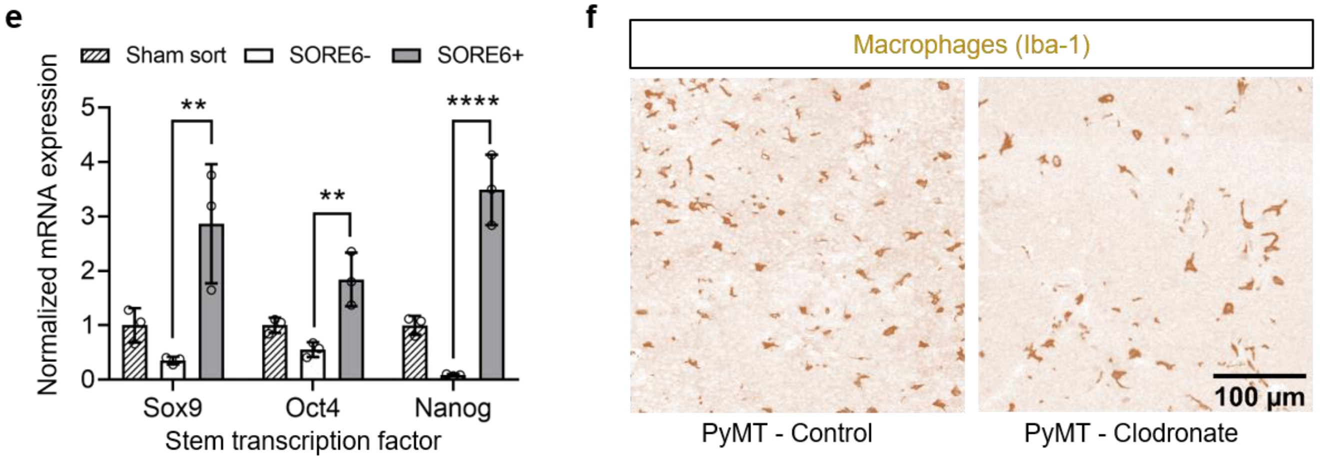

Figure S5: SORE6 reporter marks a minority cell population that is enriched in Sox9 stemness transcription factor in PyMT-derived Met-1 cells.

- **a.** Quantification of % SORE6+ cells in direct contact with macrophages or not in fixed frozen primary tumors. n=24 fields of 330x330 μm^2 in 3 mice; boxes indicate 25th-75th percentile interquartile range (IQR) and central line indicates the median. Top and bottom whiskers were plotted using Tukey method and extend to 75th percentile+1.5*IQR and 25th percentile-1.5*IQR, respectively, two-sided Wilcoxon test, ****p <0.0001.
- **b.** Non-normalized data for the CSC count (GFP-SORE6+ cells) for each day in the control and clodronate treated mice. This data was used to generate the normalized plot shown in Fig 3e.

c. Clodronate does not have a direct effect on total tumor cells, cancer stem cells (or the SORE 6 reporter) during 48h exposure in vitro. Tumor cell cultures were treated with the indicated concentrations of clodronate and total tumor cell (tdTomato positive: left panel) and cancer stem cell (SORE6>GFP+: right panel) numbers were monitored continuously by live cell imaging. Results are mean ± SD (n=3) for each time point.

d. FACS analysis showing that the SORE6 reporter identifies a minority population in cultures of Met-1 cells. SSC-A, side scatter.

e. Quantitative RT-PCR to assess the expression of master stem cell transcription factors, in FACS-sorted SORE6+, SORE6- and sham-sorted Met-1 cells. All values were normalized to the Sham sort mean and results are plotted as mean ± SD (n=3). Two-sided Tukey's multiple comparisons test; p-values: Sox9 ** = 0.0082, Oct4 ** = 0.005 and Nanog **** <0.0001.

f. Images of PyMT primary tumor tissues treated with either PBS liposomes (control) or clodronate liposomes and stained with Iba1 IHC (brown) to visualize macrophages. Similar results were obtained in n=5 mice (control) and 4 mice (clodronate) independently.

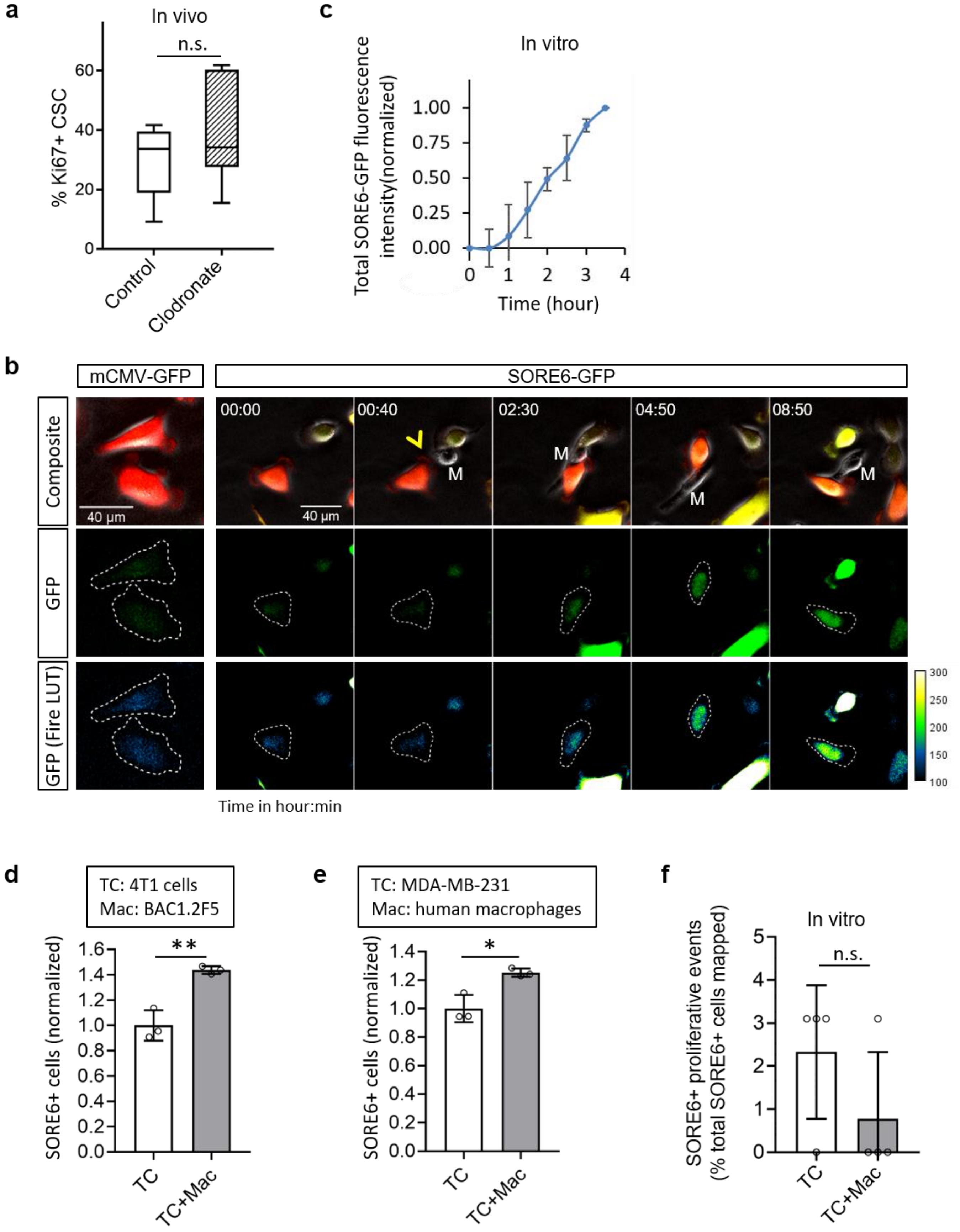
a
In vivo
n.s.
% Ki67+ CSC
0
20
40
60
Control
Clodronate
c
In vitro
Total SORE6-GFP fluorescence intensity(normalized)
0.00
0.25
0.50
0.75
1.00
0
1
2
3
4
Time (hour)
b
mCMV-GFP
SORE6-GFP
Composite
GFP
GFP (Fire LUT)
40 μm
00:00
00:40
02:30
04:50
08:50
M
300
250
200
150
100
Time in hour:min
d
TC: 4T1 cells
Mac: BAC1.2F5
SORE6+ cells (normalized)
**
TC
TC+Mac
e
TC: MDA-MB-231
Mac: human macrophages
*
f
In vitro
SORE6+ proliferative events (% total SORE6+ cells mapped)
n.s.

Figure S6: Macrophage contact induces stemness (SORE6 positivity) in multiple breast cancer cell lines

- **a.** Quantification of % CSCs that are Ki67+ in PyMT primary tumor tissue treated with either PBS liposomes (control) or clodronate liposomes and stained with Sox9 and Ki67 antibodies. n= 11 (control) and 7 (clodronate), 2-3 mm^2 fields from 5 mice for each case; unpaired two-tailed Student's t-test, n.s. p =0.148. The boxes indicate 25th-75th percentile interquartile range (IQR) and central line indicates the median. Top and bottom whiskers were plotted using Tukey method and extend to 75th percentile+1.5*IQR and 25th percentile-1.5*IQR, respectively.
- **b.** Panels from Movie 12 showing macrophage contact inducing stemness in a non-stem tumor cell in *in vitro* co-culture. Composite panel (green + red + gray phase channels): a non-stem cancer cell (red volume marker td-tomato) transitioning to a CSC (yellow) upon BAC1.2F5 macrophage (gray cell, marked with white M) contact. Yellow arrowhead points to the moment of macrophage contact with the non-stem cancer cell. Middle (GFP) and bottom panels (GFP, Fire LUT) show GFP signal increase in the tumor cell more clearly. White dotted lines mark the outline of the cancer cell. Left panels show cells expressing volume marker tdTomato and mCMV-GFP control construct, which was used to set the GFP threshold in SORE6-GFP cells.
- **c.** Quantification of total SORE6-GFP fluorescence intensity during stemness induction in tumor cells in vitro in the tumor cell-macrophage co-culture assay. Time 0 corresponds to the time when macrophage contacts the tumor cell. n=105 cells analyzed over 3 independent experiments to identify tumor cells contacting macrophages and showing induced stemness, each data point plotted as mean ± SD. Data normalized to SORE6-GFP value before macrophage contact to set the 0 background and max SORE6-GFP value as 1 after macrophage contact.
- **d.** In vitro co-culture of 4T1 tumor cells with or without BAC1.2F5 macrophages. Tumor cells were co-cultured with macrophages for 4 days before FACS measurements were made. Data plotted as mean ± SD (n=3), and normalized to the no macrophage condition, unpaired two-tailed Student's t-test, **p =0.0037.
- **e.** In vitro co-culture of MDA-MB-231 tumor cells with or without human macrophages. Tumor cells were co-cultured with macrophages for 2 days before FACS measurements were made. Data plotted as mean ± SD (n=3), and normalized to the no macrophage condition, unpaired two-tailed Student's t-test, *p =0.012.
- **f.** Quantification of SORE6+ cell proliferation due to division of pre-existing SORE6+ cells without or with macrophage contact in TC alone or TC+Mac co-culture assay respectively, using origin mapping of SORE6+ cells in time-lapse movies. Data plotted as mean ± SD, unpaired two-tailed Student's t-test, n.s. p=0.207, n=4 wells/condition for a total of 128 cells traced/condition.

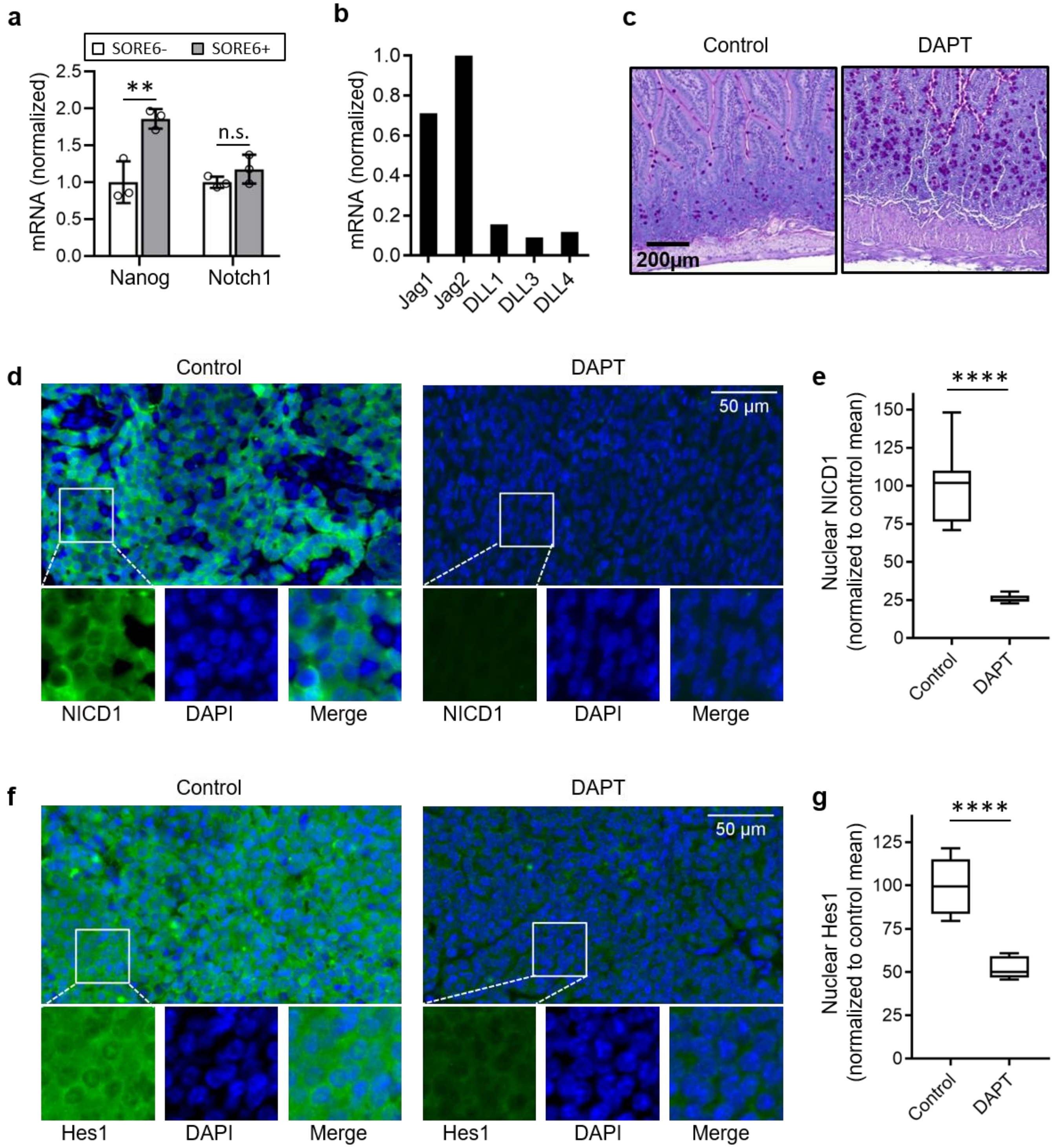

Figure S7: DAPT inhibits Notch signaling in vivo.

- **a.** MDA-MB-231 cells were sorted into SORE6+ and SORE6- populations and Notch1 expression was assessed by QRT-PCR. Data are normalized to expression in the SORE6- compartment. Nanog is the positive control. Results are mean ± SD (n=3), two-tailed Student's t-test, **p = 0.009, n.s. p =0.217.
- **b.** Notch ligands expression in BAC1.2F5 macrophages assessed by microarray. All values normalized to Jag2.

c. Periodic acid-Schiff (PAS) staining of the duodenum from MMTV-PyMT mice after 14 days of treatment with either vehicle control (left panel) or DAPT (right panel). The data confirm that in vivo DAPT-mediated Notch signaling suppression induces goblet cell (dark purple dots) differentiation. Similar results were obtained in n=5 mice (Control) and 6 mice (DAPT) independently.

d. Representative images of activated Notch1 (NICD1) staining in control and DAPT treated PyMT primary tumor tissues. Zoomed areas of the tissue are shown to highlight nuclear NICD1 localization.

e. Quantifcation of nuclear NICD1 staining in fixed PyMT mammary tumor after control or DAPT treatment. n= 8 (control) and 8 (DAPT) fields of 1-8 mm^2 tumor tissue from 2 mice (control) and 2 mice (clodronate); unpaired two-tailed Student's t-test, ****p <0.0001.

f. Representative images of Hes1 staining in control and DAPT treated PyMT primary tumor tissues. Zoomed areas of the tissue are shown to highlight nuclear Hes1 localization.

g. Quantifcation of nuclear Hes1 staining in fixed PyMT mammary tumor after control or DAPT treatment. n= 8 (control) and 8 (DAPT) fields of 1-8 mm^2 tumor tissue from 2 mice (control) and 2 mice (clodronate); unpaired two-tailed Student's t-test, ****p <0.0001.

e, g The boxes indicate 25th-75th percentile interquartile range (IQR) and central line indicates the median. Top and bottom whiskers were plotted using Tukey method and extend to 75th percentile+1.5*IQR and 25th percentile-1.5*IQR, respectively.

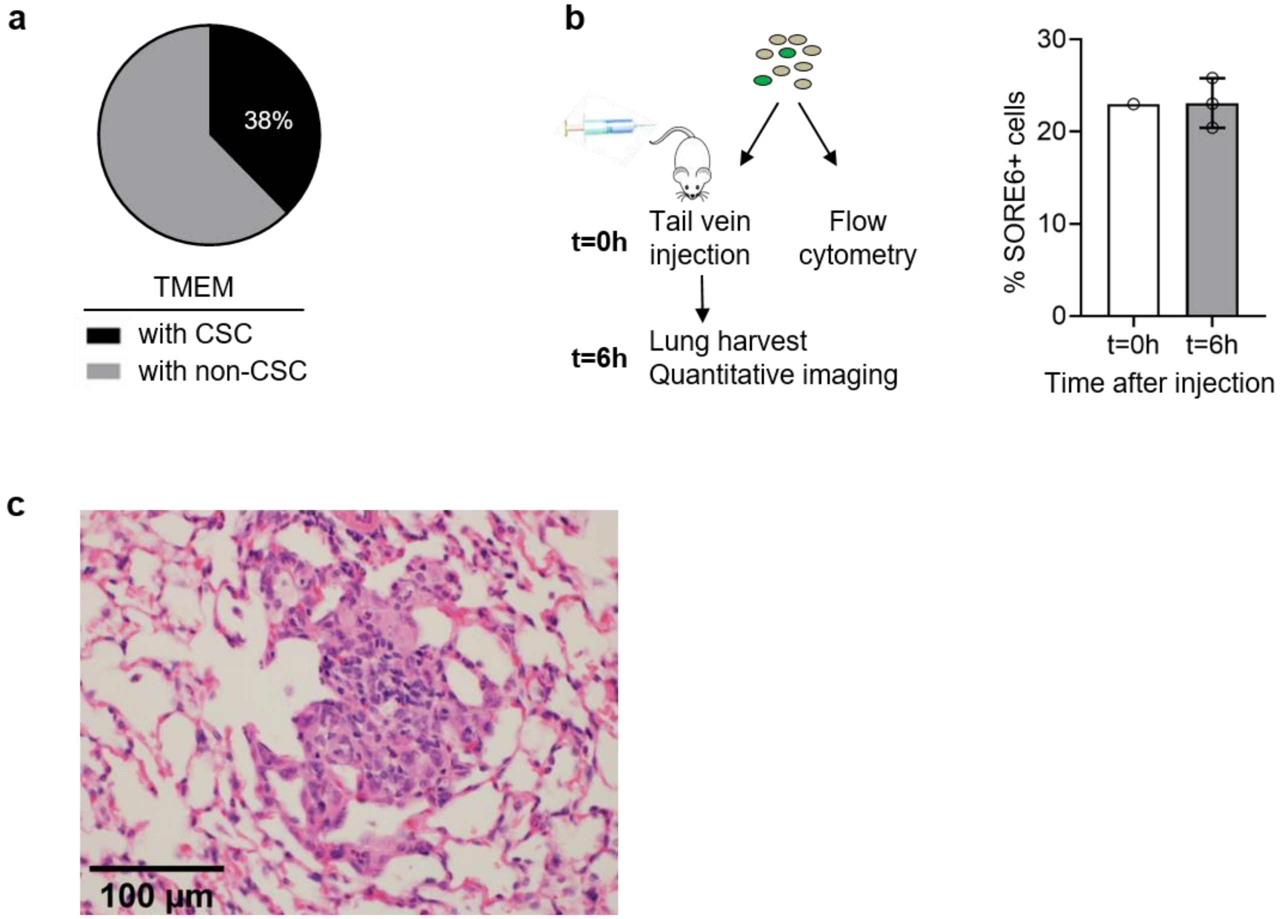

Figure S8: Additional data for Figures 6 and 7

- **a.** Frequency analysis of TMEM doorways containing CSC (SORE6+) and non-CSC (SORE6-) in SORE6>GFP xenograft MDA-MB-231 primary tumors. n=4 fields of 2.2x2.2 mm^2 from 4 mice.
- **b.** No selective die-off or interconversion of SORE6+ and SORE6- cells in the 6h between tail-vein injection and early lung colonization. The % SORE6+ cells in MDA-MB-231 model was assessed by flow cytometry immediately prior to tail vein injection into mice and by quantitative imaging of excised lungs at 6h post-injection. The gating strategy for SORE6+ cells was exactly as in figures S1g,h, namely to gate on the minCMV>GFP control construct. n=3 mice for post-injection samples, data plotted as mean ± SD.
- **c.** Detection of micrometastatic foci in the lungs of DAPT treated mice. An example of histologically detectable metastases in the lungs of SCID mice bearing SORE6>GFP MDA-MB-231 tumors is shown. Similar results were obtained in n=7 mice (control) and 6 mice (DAPT) independently.

Supplementary Table 1: Primer pair sequences for qRT-PCR of stem cell transcription factors

Human:

NANOG F:	5’-GTTCTGTTGCTCGGTTTTCT
NANOG R:	5’-TCCCGTCTACCAGTCTCACC
OCT4/POU5F1 F:	5’-AGTCTGGGCAACAAAGTGAGA
OCT4/POU5F1 R:	5’-AGAAACTGAGGAGAAGGATG
SOX9 F:	5’-GTACCCGCACTTGCACAAC
SOX9 R:	5’-TCTCGCTCTCGTTCAGAAGTC
SOX2 F:	5’-TAAATACCGGCCCCGGCGGA
SOX2 R:	5’-TGCCGTTGCTCCAGCCGTTC
SNAI1 F:	5’-GCTGCAGGACTCTAATCCAGA
SNAI1 R:	5’-ATCTCCGGAGGTGGGATG
PPIA F:	5’-GTCAACCCCACGTGTTCTT
PPIA R:	5’-CTGCTGTCTTTGGACCTTGT

Mouse:

Nanog F:	5’- GAACGCCTCATCAATGCCTGCA
Nanog R:	5’- GAATCAGGGCTGCCTTGAAGAG
Oct4/Pou5F1 F:	5’- CAGCAGATCACTCACATCGCCA
Oct4/Pou5F1 R:	5’-GCCTCATACTCTTCTCGTTGGG
Sox9 F:	5’- CACACGTCAAGCGACCCATGAA
Sox9 R:	5’- TCTTCTCGCTCTCGTTCAGCAG
