# Supplementary material for: Live tumor imaging shows macrophage induction and TMEM-mediated enrichment of cancer stem cells during metastatic dissemination
Source: Nat Commun. 2021 Dec 15;12:7300. doi: 10.1038/s41467-021-27308-2 (PMC8674234; doi:10.1038/s41467-021-27308-2)
Supplement: Supplementary file 3 — Description of Additional Supplementary Files [file 41467_2021_27308_MOESM3_ESM.docx]

Description of Additional Supplementary Files

Title: Supplementary Movie 1

Description: Intravital microscopy movie showing slow locomotion of SORE6+ stem cells (yellow). Blue (SHG): collagen fibers Time in hour:min:sec.

Title: Supplementary Movie 2

Description: Intravital microscopy movie showing fast locomotion of SORE6- non-stem cells (red). Time in hour:min:sec.

Title: Supplementary Movie 3

Description: Intravital microscopy movie showing thin invadopodial protrusion (white arrows) from CSC (yellow) interacting with ECM fiber (blue, SHG). Time in min:sec.

Title: Supplementary Movie 4

Description: Left: Intravital microscopy movie of live breast carcinoma stem cell (yellow) showing invadopodial protrusions directed towards blood vessel (gray). Blood channel has been averaged to eliminate fluctuations in intensity due to passing erythrocytes and contrast agent clearance. Right: Processed movie with the edge filter clearly shows dynamic protrusions directed towards the blood vessel. Time in hour:min:sec

Title: Supplementary Movie 5

Description: 3D reconstruction of CSC (yellow) and blood vessel (white) from Movie 4 with rotated views show invadopodium protrusion directed toward the blood vessel.

Title: Supplementary Movie 6

Description: Left: Intravital microscopy movie of SORE6+ CSC (yellow) away from the blood vessel showing dynamic invadopodial protrusions coming out in all directions. Right: Processed movie with the edge filter clearly shows the dynamic invadopodial protrusions in all directions. Time in hour:min:sec

Title: Supplementary Movie 7

Description: Non-stem cells (SORE6-) away from the blood vessel showing no cellular protrusion. (A, B) Red: tdTomato expressing and SORE6- non-stem cells; white: thresholded movie (C-E) To investigate cellular protrusions in non-stem cells, the overall translation of the cell was subtracted from each frame, so the cells appear stationary. The movie shows absence of fine cellular protrusions in non-stem cells. (E) Movie processed with edge filter.

Title: Supplementary Movie 8

Description: Left: Non-stem cells (SORE6-) close to blood vessel. Right: Movie processed with edge filter show the absences of cellular protrusion in a non-stem cell close to blood vessel.

Title: Supplementary Movie 9

Description: A montage of original (left) and drift-corrected (right) intravital movies of live breast tumor tissue showing stem cells (green), non-stem cells (red) and collagen fibers (blue). Time in hour:min:sec. Tissue drift after post-acquisition drift correction is minimal (~ 0.02 µm/min, Fig S4e) and does not contribute much to the cell migration on the time scale of the movies.

Title: Supplementary Movie 10

Description: Intravital movie of macrophage (cyan) touching a non-stem cell. After contact, stemness is induced in the in cancer cell *in vivo* as determined by the increase in GFP signal (green) over time. White dotted lines show tumor cell outlines before and after stemness induction and is drawn based on the tdTomato volume marker channel (shown on the right). Collagen fibers are shown in blue (SHG). Time in hour:min:sec.

Title: Supplementary Movie 11

Description: Intravital movie 10 shown with multiple Z-planes (each 5 µm apart), showing the induction of stemness in a non-stem cell. Note that the cell changes its color from red (tdTomato) to yellow (tdTomato+GFP). White dotted lines show tumor cell outlines in different Z-planes before and after stemness induction and is drawn based on the tdTomato volume marker channel (top panels). Collagen fibers are shown in blue (SHG). Time in hour:min:sec.

Title: Supplementary Movie 12

Description: Time-lapsed movie showing macrophage-induced stemness in a non-stem tumor cell in the *in vitro* tumor cell-macrophage co-culture assay. Macrophages (unlabeled in gray in the rightmost panel) are added to tumor cells in culture on the imaging stage at time 00:20 and one macrophage can be seen to touch a non-stem tumor cell at time 00:40. After contact, stemness is induced in the cancer cell as determined by the increase in GFP signal (green) over time (second panel). GFP channel with Fire LUT (third panel) is added to show the GFP signal increase over time more clearly. White dotted lines in the second and third panels mark the outline of the cancer cell transitioning from non-stem to stem. Time in hour:min

Title: Supplementary Movie 13

Description: Intravital movie of SORE6>GFP xenograft MDA-MB-231 tumor in Rag2 KO mice showing CSCs (yellow: tdTomato volume marker + SORE6-GFP or green: SORE6-GFP only) enriched in perivascular regions and in contact with perivascular macrophages (cyan). Blood vessels are labeled with far-red quantum dots (gray) via a tail-vein catheter. Fluctuations in blood vessel intensity are due to passing erythrocytes and contrast agent clearance. Blood vessel channel was used to outline vessels with dotted lines in all the frames. Time in hour:min

Title: Supplementary Movie 14

Description: *In vivo* intravital movie of a single SORE6+ stem cell (green) intravasating into the blood vessel in primary tumor. Macrophages are shown in cyan. Blood vessels were labeled with tail-vein injection of far-red quantum dots (shown in gray), and outlined in white dotted line.
