# Supplementary material for: Live tumor imaging shows macrophage induction and TMEM-mediated enrichment of cancer stem cells during metastatic dissemination
Source: Nat Commun. 2021 Dec 15;12:7300. doi: 10.1038/s41467-021-27308-2 (PMC8674234; doi:10.1038/s41467-021-27308-2)
Supplement: Supplementary file 18 — Reporting Summary [file 41467_2021_27308_MOESM18_ESM.pdf]

nature research

Corresponding author(s): Ved P Sharma

Last updated by author(s): Sep 15, 2021

Reporting Summary

Nature Research wishes to improve the reproducibility of the work that we publish. This form provides structure for consistency and transparency in reporting. For further information on Nature Research policies, see our Editorial Policies and the Editorial Policy Checklist.

Statistics

For all statistical analyses, confirm that the following items are present in the figure legend, table legend, main text, or Methods section.

n/a	Confirmed	
☐	☒	The exact sample size (*n*) for each experimental group/condition, given as a discrete number and unit of measurement
☐	☒	A statement on whether measurements were taken from distinct samples or whether the same sample was measured repeatedly
☐	☒	The statistical test(s) used AND whether they are one- or two-sided<br>*Only common tests should be described solely by name; describe more complex techniques in the Methods section.*
☒	☐	A description of all covariates tested
☐	☒	A description of any assumptions or corrections, such as tests of normality and adjustment for multiple comparisons
☐	☒	A full description of the statistical parameters including central tendency (e.g. means) or other basic estimates (e.g. regression coefficient) AND variation (e.g. standard deviation) or associated estimates of uncertainty (e.g. confidence intervals)
☐	☒	For null hypothesis testing, the test statistic (e.g. *F*, *t*, *r*) with confidence intervals, effect sizes, degrees of freedom and *P* value noted<br>*Give P values as exact values whenever suitable.*
☒	☐	For Bayesian analysis, information on the choice of priors and Markov chain Monte Carlo settings
☒	☐	For hierarchical and complex designs, identification of the appropriate level for tests and full reporting of outcomes
☐	☒	Estimates of effect sizes (e.g. Cohen's *d*, Pearson's *r*), indicating how they were calculated

Our web collection on statistics for biologists contains articles on many of the points above.

Software and code

Policy information about availability of computer code

Data collection	Custom-built multiphoton microscope acquisition software, Version 18, PMID: 21959234<br>Pannoramic scanner, Version 1.22.0.65408<br>SoftWoRx 7.0<br>BD Biosciences, FACS Diva Version 8<br>Adobe Photoshop, Version 2015.1.2
Data analysis	GraphPad Prism 7<br>Excel 2016<br>ImageJ 1.52s<br>VisioPharm 2018.9.0.4813<br>For custom ImageJ macro for the CSC distance analysis, source code and instructions for installation and use provided with the revised manuscript in a .zip file.

For manuscripts utilizing custom algorithms or software that are central to the research but not yet described in published literature, software must be made available to editors and reviewers. We strongly encourage code deposition in a community repository (e.g. GitHub). See the Nature Research guidelines for submitting code & software for further information.

Data

Policy information about availability of data

All manuscripts must include a data availability statement. This statement should provide the following information, where applicable:

- Accession codes, unique identifiers, or web links for publicly available datasets
- A list of figures that have associated raw data
- A description of any restrictions on data availability

No datasets were generated or analyzed during the current study.

Field-specific reporting

Please select the one below that is the best fit for your research. If you are not sure, read the appropriate sections before making your selection.

[x] Life sciences [ ] Behavioural & social sciences [ ] Ecological, evolutionary & environmental sciences

For a reference copy of the document with all sections, see nature.com/documents/nr-reporting-summary-flat.pdf

Life sciences study design

All studies must disclose on these points even when the disclosure is negative.

Sample size	Power calculations for sample size were not done a priori. For in vivo experiments, the rationale for analyzing multiple cells/fields from 3-10 biologically independent mice in each group (exact numbers mentioned in figure legends) was based on a resource efficient strategy common across relevant publications (Jiang et al. 2020 Nat Commun, PMID: 33046710; Harney et al. 2015 Cancer Disc, PMID: 26269515; Karagiannis et al. 2017 Sci Transl Med, PMID: 28679654). For in vitro experiments, cells/fields measurement were made in at least 3 biological replicates and is also common across relevant publications (Jiang et al. 2020 Nat Commun, PMID: 33046710; Pignatelli et al. 2016 Sci Rep, PMID: 27901093; Cabrera et al. 2018 Breast Cancer Res, PMID: 29636067; Tao et al. 2020 J Exp Clin Cancer Res, PMID: 32943090).
Data exclusions	No data were excluded from the analyses.
Replication	All experiments were performed with a minimum of 3 biological replicates. All experimental findings were reproduced.
Randomization	In all animal experiments involving group allocation (e.g. control vs drug treatment), mice were randomly assigned into different groups. For all in vitro experiments involving group allocation (e.g. control vs drug treatment), cells from common culture dishes were split into the different groups. None of the experiments performed on patient samples involved allocation into groups.
Blinding	No blinding as the same investigator who performed an experiment, also analyzed the data. However, to remove user-bias, same image acquisition parameters (excitation intensity, exposure time, ND filter) were used across all groups in an experiment. For SORE6 biosensor imaging, mCMV was used in every experiment to set the GFP background threshold. And automated image analysis methods/settings (e.g. threshold) were used across all samples in an experiment.

Reporting for specific materials, systems and methods

We require information from authors about some types of materials, experimental systems and methods used in many studies. Here, indicate whether each material, system or method listed is relevant to your study. If you are not sure if a list item applies to your research, read the appropriate section before selecting a response.

Materials & experimental systems

n/a	Involved in the study	
[ ]	[x]	Antibodies
[ ]	[x]	Eukaryotic cell lines
[x]	[ ]	Palaeontology and archaeology
[ ]	[x]	Animals and other organisms
[x]	[ ]	Human research participants
[x]	[ ]	Clinical data
[x]	[ ]	Dual use research of concern

Methods

n/a	Involved in the study	
[x]	[ ]	ChIP-seq
[ ]	[x]	Flow cytometry
[x]	[ ]	MRI-based neuroimaging

Antibodies

Antibodies used	rat anti-FLAG, BioLegend, cat# 637301, clone L5, PMID: 18054954<br>chicken anti-GFP, Novus Biologicals, cat# NB100-1614, PMID: 23799397<br>rabbit anti-Sox9, EMD Millipore, cat# ab5535<br>mouse anti-Cortactin, Abcam, cat#ab33333, clone 4F11, PMID: 24206842

rabbit anti-Tks5, Santa Cruz, cat#sc-30122, clone M-300, PMID: 24206842
rabbit cleaved collagen antibody, C1,2C (Col 2 3/4Cshort), Ibex Pharmaceuticals, cat# 50-1035, PMID: 22389406
mouse anti-pan Mena, BD Biosciences, cat#610693, PMID: 28679654, 19318480
Rabbit anti-Iba1, Wako, cat#019-19741, PMID: 28679654, 19318480
Rat anti-Endomucin, Santa Cruz, cat#sc-65495, PMID: 28679654, 19318480
Mouse anti-human CD68, Dako cat# M0876, PMID: 28679654, 19318480
Mouse anti-human CD31, Dako cat#M0823, PMID: 28679654, 19318480
Chicken anti-MenaINV, custom made in-house, PMID: 27901093
rabbit anti-Notch1, Cell Signaling, cat# 3608, clone D1E11
rabbit anti-Notch2, Cell Signaling, cat# 5732, clone D76A6
mouse anti-GAPDH, Abcam, cat# ab8245, clone 6C5
APC-lineage antibody cocktail, BD Pharmingen, Cat# 51-9003632, PMIDs: 9396603, 9169840, 1281687, 2898810, 1974864
Brilliant Violet 421TM anti-human CD133, Biolegend Cat#372807, PMID: 20674577
rabbit anti-Cleaved Notch1 (Val1744) (D3B8), Cell Signaling, cat# 4147
rabbit anti-Hes1, Millipore, cat# AB5702
Purified Armenian Hamster IgG Isotype control antibody, BioLegend, clone: HTK888, cat# 400901, PMID: 30590044
anti-mouse CD339 (Jagged 1) antibody, BioLegend , clone: HMJ1-29, cat# 130902, PMID: 17947672
anti-mouse Jagged 2 antibody, BioLegend , clone: HMJ2-1, cat# 131001, PMID: 18381350

Validation

Custom made chicken MenaINV antibody was validated in house (PMID: 27901093).

For rabbit anti-Sox9 antibody, EMD Millipore mentions on its website - "Anti-Sox9 Antibody is a well characterized affinity purified Rabbit Polyclonal Antibody that reliably detects Transcription Factor Sox-9. This highly published antibody has been validated in IHC & WB."

For rabbit anti-Notch1 antibody, Cell Signaling website lists 77 references for its use in a WB application such as PMID: 33976158
For rabbit anti-Notch2 antibody, Cell Signaling website lists 24 references for its use in a WB application such as PMID: 31671073
For mouse anti-GAPDH antibody, Abcam website says - "This antibody detects a band of approximately 36 kDa (predicted molecular weight: 40.2 kDa)."
For rabbit anti-Cleaved Notch1 antibody, Cell Signaling website lists following reference for its use in an IHC-IF application: PMID: 29853617
For rabbit anti-Hes1 antibody, Millipore lists IHC as one of the application and says "This Anti-HES-1 Antibody is validated for use in IHC, WB for the detection of HES-1."

Citations reporting validation/use of other antibodies are mentioned with PMID numbers in the box above.

Eukaryotic cell lines

Policy information about cell lines

Cell line source(s)

MDA-MB-231, ATCC
MDA-MB-231-LM2, Dr. Joan Massague, Sloan 720 Kettering Institute, PMID: 1604980, not commercially available
Met-1, Dr. Alexander Borowsky, University of California, Davis, PMID: 16132578, not commercially available
4T1, Dr. Fred Miller, Karmanos Institute, Detroit, PMID: 1540948, commercially available from ATCC
BAC1.2F5, Dr. Richard Stanley, Albert Einstein College of Medicine, PMID: 3031090, not commercially available
HUVEC, Lonza, Walkersville, MD, USA Lot # 0000396930
human primary monocyte-derived macrophages, StemExpress, Folsom, CA, Cat# PBMAC001.5C
Control and Jag1KO BAC1.2F5 macrophages, PMID: 29636067, not commercially available

Authentication

Authentication of the human cell lines was performed using the Promega Powerplex 16 system for STR allele typing. The assay was performed by Laragen Corp. The STR allele were searched against the ATCC database for match to the known STR profile for MDAMB231. The 4T1 and Met-1 mouse cells were obtained from the originating investigators, and used within 10 passages of freezing. Their morphology and biological properties at the time of assay were consistent with those on original receipt of the cell lines. STR profiling of mouse cells only reveals the background strain of the originating mouse and will not distinguish tumor cells derived from the same inbred strain.

Mycoplasma contamination

All cell lines tested negative for mycoplasma contamination.

Commonly misidentified lines
(See ICLAC register)

None

Animals and other organisms

Policy information about studies involving animals; ARRIVE guidelines recommended for reporting animal research

Laboratory animals

ECFP macrophage/Rag2 knockout mice (Rag2KO Macblue) were generated from the crossing B6.129S6-Rag2tm1Fwa N12(Rag2 - Model RAGN12, Taconic) with 804 Tg(Csf1r*-GAL4/VP16,UAS-ECFP)1Hume/J (Stock No: 026051, the Jackson Laboratory).
Species: Mus Musculus, Strain: C57BL/6, Sex: Female, Age: 14-16 weeks

NCI SCID/NCr mice, female, 6 weeks old (strain code: 561, Charles River)

PyMT mice, Species: Mus Musculus, Sex: female, Strain: FVB, Age: 8-10 weeks

For all mice, the light cycle in the room was set for lights on at 6am and off at 8pm. Ambient temp was kept at 72 +/- 4 deg F, 35% relative humidity.

Wild animals: study did not involve wild animals

Field-collected samples: study did not involved samples collected from the field

Ethics oversight: All procedures were conducted in accordance with the National Institutes of Health regulation concerning the care and use of experimental animals and with the approval of Albert Einstein College of Medicine Animal Care and Use Committee (IACUC).

Note that full information on the approval of the study protocol must also be provided in the manuscript.

Flow Cytometry

Plots

Confirm that:

☒ The axis labels state the marker and fluorochrome used (e.g. CD4-FITC).

☒ The axis scales are clearly visible. Include numbers along axes only for bottom left plot of group (a 'group' is an analysis of identical markers).

☒ All plots are contour plots with outliers or pseudocolor plots.

☒ A numerical value for number of cells or percentage (with statistics) is provided.

Methodology

Sample preparation: For cultured cells, cells were typsinized, pelleted, and resuspended in 2% BSA/PBS as a single cell suspension for FACS sorting. Tissues from primary tumors were washed once with PBS, minced, suspended in PBS, passed through a 70µm cell strainer, and centrifuged down to cell pellets. Pellets were resuspended in PBS, filtered with a 40µm cell strainer, spun down, resuspended in 200µL of 2% BSA/PBS, and stained with APC-linage antibody cocktail (BD Pharmingen, APC mouse lineage antibody cocktail, Cat# 51-9003632) at 5µL antibody/million cells for 30 minutes at room temperature. After staining, the cells were washed twice with PBS and resuspended in 2% FBS/PBS for FACS sorting.

Instrument: Flow cytometry analysis was performed using an LSRFortessa SORPI (BD Biosciences) and FACS sorting using an BD FACSAria Fusion

Software: Data analysis was performed using FlowJo_v10.6.2 (Tree Star, Ashland, OR, USA) software.

Cell population abundance: The purity of FACS-sorted samples was assessed by post-sort flow cytometry. All sorted fractions had a purity of 80-95%.

Gating strategy: FSC and SSC gates were used to select for live single cells from the starting population. Tumor cells were identified as cells in the APC-Lineage negative gate, defined using the APC-isotype control. Cells expressing the control minCMV>dsCopGFP construct were then used to establish the GFP gate that was used for the SORE6>dsCopGFP expressing cells. For cytometry, the GFP gate was set so that >99.9% of the control cells fell in the negative gate. This gate was then used to define SORE6+ vs SORE6- cells.

☒ Tick this box to confirm that a figure exemplifying the gating strategy is provided in the Supplementary Information.
